# A Comprehensive Study of Data Collection Schemes Using Mobile Sinks in Wireless Sensor Networks

**DOI:** 10.3390/s140202510

**Published:** 2014-02-05

**Authors:** Abdul Waheed Khan, Abdul Hanan Abdullah, Mohammad Hossein Anisi, Javed Iqbal Bangash

**Affiliations:** Faculty of Computing, Universiti Teknologi Malaysia (UTM), Johor 81310, Malaysia; E-Mails: wkabdul2@live.utm.my (A.W.K.); anisi@utm.my (M.H.A.); ibjaved2@live.utm.my (J.I.B.)

**Keywords:** sink mobility, energy consumption, data delivery latency, packet delivery ratio, wireless sensor networks

## Abstract

Recently sink mobility has been exploited in numerous schemes to prolong the lifetime of wireless sensor networks (WSNs). Contrary to traditional WSNs where sensory data from sensor field is ultimately sent to a static sink, mobile sink-based approaches alleviate energy-holes issues thereby facilitating balanced energy consumption among nodes. In mobility scenarios, nodes need to keep track of the latest location of mobile sinks for data delivery. However, frequent propagation of sink topological updates undermines the energy conservation goal and therefore should be controlled. Furthermore, controlled propagation of sinks' topological updates affects the performance of routing strategies thereby increasing data delivery latency and reducing packet delivery ratios. This paper presents a taxonomy of various data collection/dissemination schemes that exploit sink mobility. Based on how sink mobility is exploited in the sensor field, we classify existing schemes into three classes, namely path constrained, path unconstrained, and controlled sink mobility-based schemes. We also organize existing schemes based on their primary goals and provide a comparative study to aid readers in selecting the appropriate scheme in accordance with their particular intended applications and network dynamics. Finally, we conclude our discussion with the identification of some unresolved issues in pursuit of data delivery to a mobile sink.

## 1. Introduction

**Figure 1.**
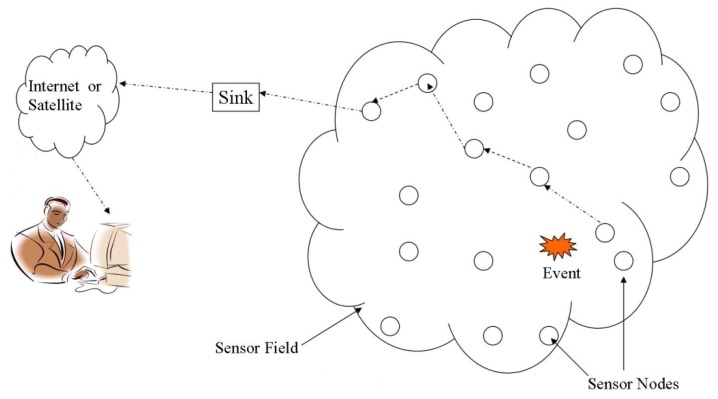
Wireless sensors network.

In recent years, wireless sensor networks (WSNs) have seen tremendous applications in different aspects of our lives such as habitat, structure health and remote health monitoring, precision agriculture, home automation, smart electric grids, and intelligent transportations systems. Typically, a large number of tiny computing devices (nodes) constitute a WSN where nodes are considered as constrained in resources, *i.e.*, with limited on-board memory, short-range radio transceivers, and battery power. Depending on the application environment, nodes are interfaced with various sensors for monitoring some phenomenon of interest (temperature, humidity, pressure, *etc.*) and forward sensory data to special devices (sinks) in a cooperative manner (typically multi-hop). The sink device (base-station) upon receiving the sensory data analyses the reported activity and may further route the data to a remote user/database via some regular infrastructure such as the Internet [1]. A typical WSN architecture is illustrated in Figure 1.

Nodes in a sensor network are battery operated and in most situations, battery replacement or recharging is not viable. To achieve prolonged network lifetime, sensor nodes must tailor their activities in an energy-efficient way so that the scarce energy reserves are used very efficiently. Upon deployment, sensor nodes sense, process and communicate an observed phenomenon. Among these tasks, communication is considered as the main consumer of sensor energy reserves, thereby imposing strict energy-aware constraints on all communication activities by the sensor nodes [2]. Since routing protocols and media access control (MAC) protocols are directly related to the communication module, hence protocols at these two layers must make an intelligent utilization of the scarce energy resources.

For prolonged network lifetime, not only is the energy consumption of individual sensor nodes important, but also balanced energy consumption among all the sensor nodes is desired [3]. In traditional WSNs, sensor nodes are distributed in the sensing field whereupon detecting some event of interest, nodes report the sensed event back to some static sink(s) through multi-hop or single hop communication. One major drawback of such communication infrastructures is that the sensor nodes close to the sink will consume more energy (partly for reporting their own sensed data and partly for relaying their neighbors' data), and thus their energy will deplete quickly. Consequently, this will result in isolation of the sink and as a whole the entire network would no longer be operational. This problem is commonly known as the hot-spot or sink-hole problem in wireless communication. To deal with this issue, the concept of mobile sink was introduced in [4,5], that not only results in balanced energy consumption among the nodes but can also be exploited to connect isolated segments of the network [6]. Another motivation for introducing a mobile sink in a WSN is that some applications explicitly require sink mobility in the sensor field. For instance, a rescuer equipped with a PDA moves around in a disaster area to look for any survivors [7], and a farmer while walking around a field would be interested in knowing which segment of the field requires watering, fertilizers, *etc.* Although the sink mobility improves network lifetime, at the same time it incurs additional overhead for the routing protocol for dynamic route adjustments. Due to sink mobility, the topology of a WSN becomes dynamic and to cope with such a dynamic topology, the routing algorithms specifically designed for static WSNs cannot be directly applied in mobility situations. This has triggered the development of new routing strategies for Mobile sink-based Wireless Sensor Networks (mWSNs).

In this paper, sink mobility is covered from different perspectives with the main aim of critically discussing the performance of existing mobile sink-based data collection schemes. Sink mobility has also been exploited to address coverage issues and interested readers may refer to [8–11] for more details. The rest of this paper is organized as follows: first, the network architecture of mWSNs is described in Section 2. Next in Section 3, the potential advantages that are obtained by exploiting the sink mobility are discussed. Then some challenges for data dissemination that are caused by sink mobility are identified in Section 4. Different mobility patterns exhibited by sinks are discussed in Section 5, as they have a direct impact on the design of a strategy for data delivery towards a mobile sink. A procedure of data delivery to a mobile sink is described in Section 6 to gain more insight into the complexity and the different phases involved when delivering sensed data towards a mobile sink. Based on the sink mobility patterns, the existing mobile sink-based data collection schemes, which are discussed in detail along with their aims, methodologies, strengths and weaknesses in the various sub-sections, are classified into different classes in Section 7. Section 8 organizes existing schemes on the basis of their main goal(s) and provides a comparative study in terms of the various features. Finally, Section 9 concludes our discussion with identification of issues that need to be addressed in pursuit of data delivery to a mobile sink.

## 2. Network Architecture of Mobile Sink Based Wireless Sensor Network

**Figure 2.**
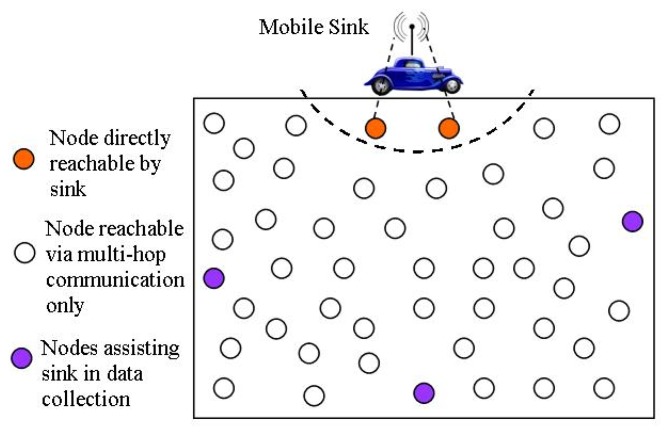
Network architecture of a mobile wireless sensor network.

The mWSN network architecture differs from that of a static WSN in the sense that in the former case, the sink keeps on moving around/inside the sensor field for efficient data collection. A reference mWSN network architecture is shown in Figure 2. The main components of a mWSN are given as follows:

*Regular Nodes—*These are the ordinary sensor nodes that are deployed in the sensor field for sensing some phenomenon of interest. Upon sensing the events, these nodes disseminate their data in a cooperative manner towards a mobile sink. Depending on their placement in the sensor field, nodes might work as relays thereby forwarding others data towards a mobile sink.
*Mobile Sink(s)—*Depending on the application scenario, there might be single/multiple mobile sink(s) that move inside/around the sensor field for data collection. Such devices are considered unconstrained devices in terms of their resources. Mobile sink can be a sensor node attached to a human, car, animal or a robot.
*(Optional) Sink Assistants—*In some applications, special nodes are deployed at strategic positions that provide assistance to the sink in data collection. These devices are also considered as energy rich. In static deployment, such nodes become intermediate/local data collectors from the sensor nodes and later on deliver collected data to a mobile sink upon its arrival. In the mobility case, they are meant to ensure coverage of almost the entire sensor field for real-time communication services in certain applications.

## 3. Sink Mobility Advantages

In almost all WSN applications, the sink is considered as an unconstrained entity in terms of resources (energy reserve, processing power, communication capability, *etc.*). Likewise, in several applications of sensor networks, sink mobility can be realized by attaching a sink device to a mobile entity such as human, animal, robot, or vehicle which can move around/inside the sensor field for data collection. Thus considerable energy savings can be obtained by deploying a mobile sink in sensor field. Kinalis *et al.* identified several potential advantages of sink mobility [12] in the sensor field that are outlined as follows:

*Sensor Lifetime Enhancement—*By exploiting sink mobility, not only is the energy-hole problem alleviated, but it also improves the lifetime of nodes thereby reducing the multi-hop communication. The sensor nodes are considered as energy constrained devices whereas the sink, being external to the network, does not have any energy constraints. Since the communication module is considered the main consumer of a node's energy reserves, if the sink moves closer to the event reporting nodes, greater energy savings could be obtained, thereby limiting the multi-hop communication.
*Improved Coverage—*A mobile sink can potentially cover sparse networks due to its mobility feature and therefore is considered as a cost-effective solution offering deployment of fewer sensor nodes in the sensor field. Similarly, due to its mobility feature, the mobile sink can also pass through problematic areas where there is some obstruction in the propagation path such as large boulders.
*Improved Throughput and Data Fidelity—*Exploiting sink mobility can also achieve improved throughput and data fidelity. If the sink moves towards the event reporting area, it will not only reduce the number of transmissions but will also reduce the probability of transmission errors and the chances of collisions. Minimizing the chances of retransmissions not only improves network throughout, but also prolongs the network lifetime.
*Improved Security—*Compared to the static sink scenario, a mobile sink poses less security threats since due to the sink mobility it is relatively difficult to overhear the information. Furthermore, an adversary can obtain the information regarding only a small area due to the reduced number of multi-hop transmissions. Similarly, possible attacks targeting the hub sensor nodes to disrupt network operation are not possible as due to sink mobility, there are no such geo-strategically important nodes that the messages must always pass through.

## 4. Sink Mobility Challenges

Although exploiting sink mobility results in several advantages, at the same time it generates more network overhead that need to be addressed by the routing protocols. In this regard, any routing protocol dealing with sink mobility needs to carry out the following additional operations [13]:

- Inform the neighbors upon link breakage with the mobile sink.
- Propagate the sink's topological updates for ensuring connectivity.
- Reduce the chances of packet loss while the sink moves from one point to another.

However, these operations cannot be taken in a holistic manner as that would greatly compromise the sensor node's energy consumption for every single move of the mobile sink. The following are the various challenges that arise due to sink mobility [14] which cannot be otherwise seen in static sink scenarios:

*Sink Contact Detection—*For communication with a mobile sink, first its presence needs to be detected in their communication range by the sensor nodes. Sink contact detection is greatly affected by the speed of the mobile sink and the nodes' duty-cycles, as nodes in sleep mode will not be able to detect the presence of a moving sink. Similarly high sink mobility results in a short contact time with the sensor nodes which consequently leads to a high packet loss ratios.
*Mobility-aware Duty Cycle Management—*To propagate a sink's topological updates, sensor nodes need to be in listening mode. If the mobility of the sink can be predicted or computed by exploiting the knowledge about the sink mobility pattern, it could help the nodes optimize the sink detection. In situations where the visiting times are known *a priori* or computable with certain accuracy, nodes' duty-cycles can be adjusted accordingly to bring them into active mode at the expected arrival time of the mobile sink. However, this is only applicable in situations where the sink always follows a certain trajectory while maintaining a constant speed.
*Increased Message Delivery Latency—*There is always a trade-off between energy consumption and message delivery latency in a mWSN. The message delivery latency directly depends on the sink mobility pattern (speed, direction, and pause interval). If the latest location information of a mobile sink together with the sink mobility information is promptly propagated in the entire network, the end-to-end latency can be reduced by enabling the nodes to adjust their routes to the sink accordingly. However, doing so would result into enormous energy consumption and would decrease the network lifetime. On the other hand, if the latest location information is propagated infrequently or only a limited number of nodes are being informed, the path adopted for data delivery may not be the optimal one in terms of hop-count. In addition, the message delivery latency is greatly influenced by the temporary pause period of the mobile sink. In some situations, the sink knocks at every single node to collect the sensed data. In such scenarios, if the size of the event data is big enough that it cannot be uploaded to the sink within the contact time, the nodes wait for the next trip of the mobile sink. However, doing so results in increased message delivery latency or overriding of buffered message by fresh sensory data.
*Increased Packet Loss Ratio—*Due to unavailability of fixed contacts with the mobile sink, data is forwarded by each sensor node towards the last known location of the mobile sink. However, if the latest mobility information is not propagated in the entire network, successful delivery of the forwarded message would be greatly compromised. This problem gets further complicated when a mobile sink significantly moves from the last known sojourn point, ultimately resulting in dropping of the message due to a long traversal time.

## 5. Sink Mobility Patterns in Wireless Sensor Networks

There are three basic mobility patterns that a mobile sink can exhibit in a sensor field [15]: random mobility, predictable/fixed-path mobility and controlled mobility. Both the random and predictable sink mobility are not in control of the network/observer.

*Random/Unpredictable Mobility Pattern—*Random mobility pattern is exhibited if the sink device is attached to a mobile unit such as an animal which has no knowledge of its mobility (speed and direction). In this type of mobility, the sink makes the next move autonomously in terms of speed and direction. This mobility pattern is being characterized as highly unpredictable regarding the future position of the sink. For delay tolerant applications, random sink mobility results in prolonged network lifetimes [16] and is particularly applicable in situations where node deployment is not known.

*Predictable/Fixed-Path Mobility Pattern—*This is the simplest among the three mobility patterns and is exhibited by the sink if it always follows a certain trajectory such as along the periphery of the sensing field. Using this mobility pattern, nodes along the trajectory can learn the expected time of visit of the mobile sink [15] and thus accordingly optimize their sensing and data delivery tasks. This type of mobility pattern is particularly appealing for applications such as car parks alongside roads where a mobile sink (car driver) can enquire about availability of parking slots in some region of interest.

*Controlled Mobility Pattern—*Controlled sink mobility refers to the situation when the observer of a sensor network can control the motion of the mobile sink [17]. Controlled mobility is exhibited by sinks if the sink device is attached to a mobile unit such as a robot. Controlled sink mobility is adopted based on the assumption that context-aware pervasive devices are at the disposal of network. The context-aware devices retrieve their possible future locations based on the data generated by the field sensors [18]. Depending on the application goal, the mobile sink adapts its movement (both speed and trajectory) in a deterministic way to achieve better results. For example, if the goal is to prolong network lifetime, the sink makes its next move towards energy rich areas to achieve a balanced utilization of nodes' energy reserve. Similarly, in applications where message delivery latency is critical, the sink moves towards event reporting areas to reduce multi-hop communications. However, the visiting schedule of the mobile sink must be carefully adopted as infrequently visiting particular network segments might result in large data delivery latency [19]. In terms of cost effectiveness, controlled sink mobility is not a preferred choice, but if the goal is to reduce the data delivery latency, controlled sink mobility yields better results [20] than random and/or fixed-path mobility options.

## 6. Procedure of Data Delivery to a Mobile Sink

In this section, we outline the different phases involved in reporting observed data to the mobile sink. Without loss of generality, we consider the reference scenario shown in Figure 2, where a mobile sink comes in contact with some field sensors that come across in its communication range. The time a node is in communication range of a mobile sink is considered as contact time and the contact area is the area in which the sink is reachable by the node within its communication range as shown by the dashed arc in Figure 2. A node can only deliver sensory data to sink if either it detects the presence of sink in its radio communication range (single-hop communication) or it knows someone who can possibly come in contact with the sink (multi-hop communication). Therefore, for data delivery to the sink, a node must go through three phases, namely sink discovery, route determination, and finally the actual data transfer [14]. These three phases are briefly discussed in the following paragraphs:

*Sink Discovery—*Due to the dynamic network topology caused by sink mobility, sink discovery is the first step towards reporting the sensed data to a sink. Since the sink keeps on changing its position, nodes need to continuously keep track of the new location of the sink if no sink-assistants are operating in the network. Sink discovery is greatly affected by nodes' duty-cycles and the speed of the sink [14]. Any node that detects the presence of sink in its communication range will report to the rest of the network about this development and will immediately start transferring the buffered data (if any) to the sink. However, the temporary contact time should be long enough so that the sink's neighbors can complete the data transfer to the sink, failure to which results in huge data delivery latency. For prompt sink discovery, some approaches [20–22] employ multiple radios: a long-range one for data communication and another short-range one for awaking nodes. The short-range radio listens to the channel continuously and activates the long-range radio upon detecting a tone from the sink. However, most of commercially available nodes do not have such dual-radio hardware support [14].
*Route Planning—*Upon discovery of the sink, the sensor nodes will determine an optimal route to reach the sink. In this regard, they can adopt multi-hop communication or can exploit cluster-based communication. The goal in routing is to reach to the sink with minimum cost and depending on the application, the cost could be energy consumption associated with the number of hops in a particular route, the data delivery latency following that route, or the reliability (in terms of packet loss ratio).
*Data Transfer—*Finally, data is delivered to the sink with the goal of ensuring maximum utilization of the contact time with the sink, thereby maximizing the throughput. The data transfer is not only affected by channel conditions, but also by the distance between source nodes and sink which keeps on changing subject to the sink's speed [14].

## 7. Classification of Mobile Sink based Data Collection Schemes

**Figure 3.**
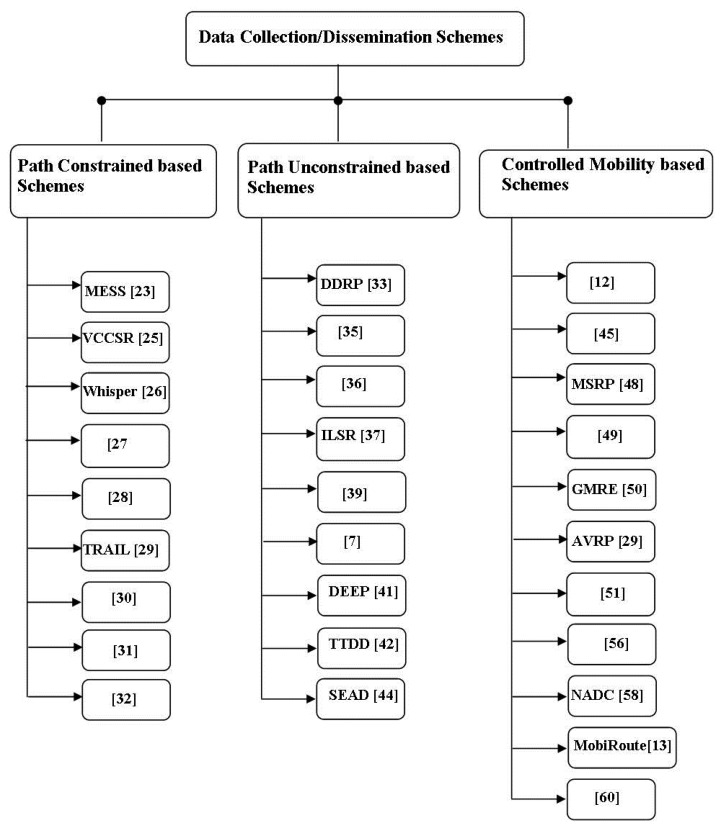
Classification of sink mobility based data collection/dissemination schemes.

In recent years, a number of data collection schemes have been proposed for mWSNs. Based on sink mobility patterns, we classify these schemes into three main categories: Path Constrained Sink Mobility-based Schemes, Path Unconstrained Sink Mobility-based Schemes, and Controlled Sink Mobility-based Schemes. Figure 3 illustrates the taxonomy of sink mobility-based data collection/dissemination schemes covered in this paper. In the following sub-sections, we briefly discuss the relevant schemes in each of these categories.

### 7.1. Path Constrained Sink Mobility-Based Schemes

In this category, we discuss those data collection schemes where the sink follows a constrained path such as a straight line, periphery, circular path, or visits only a selected subset of nodes in the sensor field for data collection. In the rest of this section, an overview of the state-of-the-art is given, including their aims, methodologies, strengths and weaknesses.

**Figure 4.**
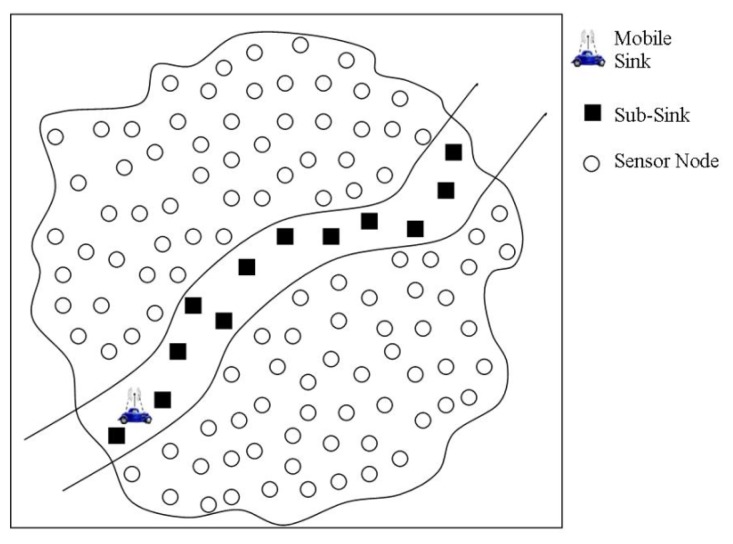
Strip-based structure in MESS.

In [23] a scheme called Multiple Enhanced Specified-deployed Sub-sinks (MESS) for WSNs with path-limited sink trajectories is proposed. A similar approach has also been adopted in [24]. MESS employs multiple sub-sinks for sensory data collection from more remote nodes. The aim is to reduce the long-haul communication between the source nodes and mobile sink and thus the delay as well. The sub-sinks are considered as enhanced wireless nodes having more storage capacity and are deployed at equal distances along the accessible path after the deployment of the rest of the network nodes. The placement of sub-sinks in this fashion creates a strip in the sensing field and provides a set of meeting points with the mobile sink as shown in Figure 4. Each sub-sink working as an access-point to the mobile sink, notifies the underlined network segment about the service that it is offering. For that purpose, each sub-sink sends a broadcast message (containing its ID) so all the recipients would set the sub-sink as the next-hop. In case if a node receives broadcasts from more than one sub-sinks, it selects its next-hop to the sink based on received signal strength. Consequently, the one-hop neighbors of sub-sinks would broadcast this route alert further in the network till all sensor nodes are informed.

In the data collection phase, MESS assumes that the speed of mobile sink is quite slow such that it collects all the harvested data from at least two of the sub-sinks along its trajectory while moving continuously. However, in large scale networks each sub-sink might be depositing data for a large population of sensor nodes and consequently that cannot be delivered to the mobile sink in a single trip. As a result, huge latency would be caused in the data delivery to the sink. Another implication is the use of specialized nodes working as sub-sinks, which not only need to be rich in storage capacity, but also in energy. These implications together with the fixed speed of mobile sink limit its applicability to certain application environments only. Similarly, nodes in the vicinity of sub-sinks would suffer from early energy-depletion thereby reducing the overall network lifetime.

**Figure 5.**
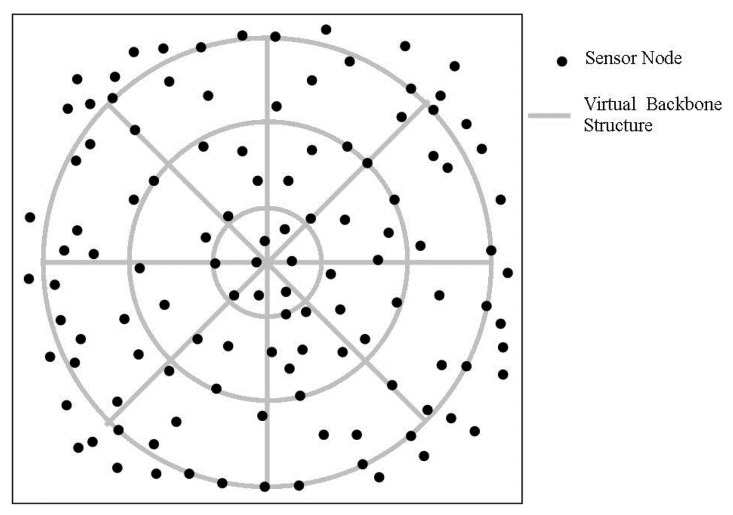
An example of virtual backbone structure in VCCSR [25].

Chen *et al.* proposed a converge-cast algorithm called Virtual Circle Combined Straight Routing (VCCSR) [25] for efficient data collection in mWSNs. VCCSR aims to reduce the path reconstruction cost upon sink mobility and for this purpose, it constructs an adjustable routing tree. In the proposed algorithm, it forms a virtual backbone structure which is comprised of several virtual circles and straight lines where Cluster-Head (CH) nodes are placed along the virtual circles and straight lines. An example virtual backbone structure is shown in Figure 5.

**Figure 6.**
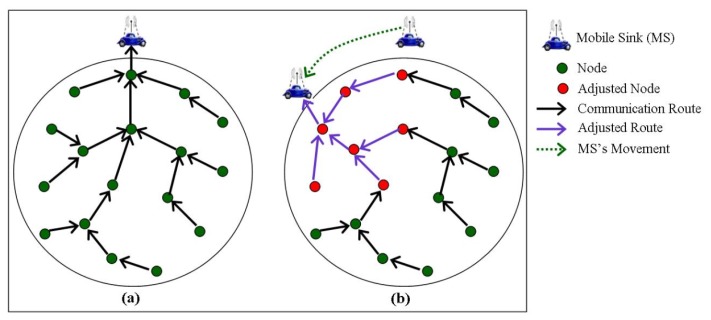
Partial tree readjustment in VCCSR upon sink mobility [25].

The CH points are computed as the midpoints of virtual paths and accordingly nodes which are closer to CH points are appointed as CH nodes. Furthermore, all nodes located at the centre of circles, the rendezvous points (intersecting points of the circles and straight lines), and the boundary points (midpoints of the routes to rendezvous points) are considered as CH nodes. Together all the CH nodes form the virtual backbone network. Similarly, the boundary points in the virtual structure are also used in deciding which nodes need to be adjusted in readjusting the routing tree upon sink mobility. CH nodes assume the responsibility of collecting sensed data from its cluster members and delivering it to the sink via a tree-based structure. For data collection, the mobile sink periodically collects data from the sensor field by forming a converge-cast tree first. To construct such a tree, the sink selects the CH node at the closest rendezvous point as the root and transmits a query to that CH node. The CH node accordingly propagates the query to sensor network via the backbone network and forms a tree structure using all CH nodes which provides shortest path from all CH nodes to mobile sink. Consequently, the CH nodes knowing location of neighbor CH nodes, and mobile sink together with the associated rendezvous point location route the sensed data using minimal hops towards the mobile sink. Upon sink mobility, VCCSR avoids reconstruction of the whole routing tree as the sink updates only the part of the tree (a few CH nodes) that need to reconstruct the routing tree as shown in Figure 6. This partial tree reconstruction yields great energy savings.

In VCCSR, the controlled reconstruction of the routing tree results in decreased energy consumption and thus helps in prolonging network lifetime. Furthermore, it also facilitates continuous data delivery to the mobile sink. However, VCCSR is not suitable for event driven applications which cannot tolerate significant delay in message delivery. In addition, it considers sink mobility along a circular path outside the sensor field only where the sink maintains communication with the border nodes. We argue that imposing the sink's communication with border nodes only, results in imbalanced energy consumption of sensor nodes as border nodes would consume more energy compared to innermost nodes. This would decrease the network lifetime caused by early depletion of border nodes' energy reserve.

In [26] Oliveira *et al.* proposed a greedy forward algorithm called Wireless HIgh SPEed Routing (*Whisper*) for sending data towards a high speed sink that aims to ensure guaranteed data delivery. It is based on the idea that the sink, due to its high speed, will not stay at the location at which it injected the query and thus it sends the response towards a more up to date location of the high speed sink. It assumes that all the nodes know their locations, neighbor's locations, and the sink's trajectory (included in query packet) and displacement. Furthermore, it assumes that the sink will not vary its speed and will follow a trajectory along a straight line. The field sensor nodes will generate the response and will forward it towards the estimated meeting point with the sink. The meeting point is estimated on the basis of considering the various delays in message transmission together with node's own location, the neighbors' locations, and the information supplied in query packet. This scheme limits its applicability by imposing two constraints, *i.e.*, the constant speed of the mobile sink and its future trajectory, which may not be realistic assumptions as in most of the cases a mobile sink keeps on changing its speed and directions.

**Figure 7.**
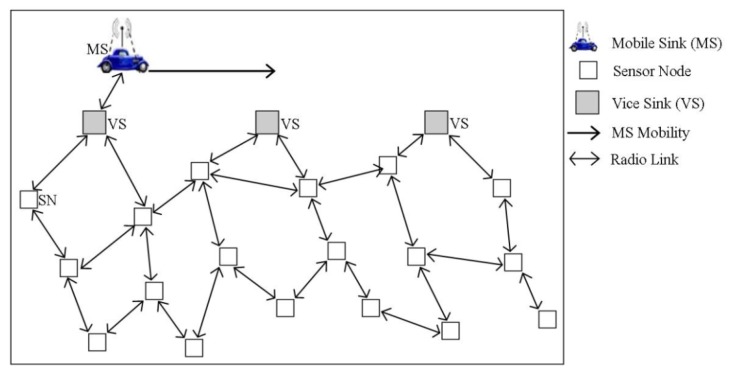
System architecture: A mobile sink (MS) queries network via vice-sinks (VSs). Sensor nodes (SNs) inside network communicate in multi-hop fashion to reach to VSs [27].

Tacconi *et al.* proposed an energy-aware and delay-aware data forwarding scheme [27] specifically tailored to incorporate WSNs in an intelligent transportation system. It follows a query driven data delivery strategy. Using this scheme, to forward a response towards a mobile sink, the traditional geographic routing is optimized such that the next hop node is selected on the basis of its relative distance to the sink and its residual energy level. In the considered scenario, as shown in Figure 7, a car injects a query (such as information about parking slots) into the sensor field via roadside nodes (vice-sinks) and consequently collects the response from one of the vice-sinks along its trajectory. The query contains information such as ID of the mobile sink, its coordinates, speed, direction, time-stamp, coordinates of target area along with radius of target area. All the nodes including the vice-sinks, are considered as location-aware and thus forward the query towards the area of interest using the geographic routing. A central node in the area of interest collects the requested information, generates a response message and based on the mobility information provided in the query packet, estimates the target location of the mobile sink. Accordingly, it forwards the response towards the anticipated location of the sink in a multi-hop manner. Finally, the response message is delivered to the nearest vice-sink. If the mobile sink has not yet arrived at the vice-sink, it waits for the mobile sink and delivers the response message upon its arrival. However, if the mobile sink has already passed the vice-sink, it routes the response message towards the next vice-sink via its one hop sensor node. This process goes on till the response message is delivered to mobile sink or is timed out and thus dropped. The proposed scheme in [27] achieves balanced use of nodes' energy consumption and latency-aware message delivery to a mobile sink. However, it does not fit well in situations where the sink trajectory is not known *a priori* or cannot be predicted.

In the scheme proposed in [28], data delivery to mobile sinks is considered for delay-tolerant applications. It aims to minimize the number of transmissions required to deliver data to mobile sinks thereby exploiting the knowledge about the likely trajectories of the mobile sinks. It exploits the fact that in certain application environments, the mobile sink is aware of its future trajectories constrained by the motion patterns like roads, trails or hallways. Hence, in the initial phase, the mobile sinks announce a set of anticipated trajectories (collected offline) to nodes in the form of a broadcast destined for the entire network. In this approach, sensed data is not directly routed to mobile sinks but rather to some relays which lie along any of one of the trajectories of the mobile sink. These relays stash the reported data and deliver to sink when the sink passes by them in the future. It also tries to accurately predict a set of stashing points covering all trajectories of the mobile sinks that can be potentially visited by sinks in the near future. Furthermore, it makes it mandatory to have at least one stashing point on each trajectory, thus ensuring that the data will not be lost and will be retrieved by at least a single mobile sink. To find the optimal stashing points, linear programming is adopted for each data source to cope with the uncertainty about the trajectory of mobile sink. This scheme achieves guaranteed data delivery to mobile sinks at the expense of more energy consumption in the form of redundant transmissions in different directions. TRAIL [29] is another lightweight routing protocol specifically designed for low traffic load mWSNs that aims to deliver data to mobile sinks with minimal protocol overhead. The proposed protocol adopts both random walk- and trail-based forwarding strategies for data delivery to mobile sinks. In situations where a node has no fresh knowledge (trail) of any sink, a packet is forwarded using a random walk strategy until it reaches a node which has fresh knowledge (trail) about a sink. From that point onwards, the packet follows the trail till it is delivered to a sink. In TRAIL, each node builds and maintains two tables: sink-table and route-table. In the sink-table, a node keeps a record of whether the node is/was a neighbor of sink together with time-stamp information whereas the route-table keeps a record of the next-hop nodes towards the sink. An integral part of the TRAIL protocol is the trail formation. A sink's trail is formed by periodic broadcasting of beacon messages where the rate of beacon messages generation is tuned taking into account the velocity and communication range of nodes. Each beacon message is comprised of a sink's ID and time-stamp information. Nodes upon receiving beacon messages update their sink-tables and do not further propagate beacon messages. Thus, a trail is being formed from all those nodes which have been recently visited by a mobile sink. Consequently, this trail is used for data packet forwarding by sensor nodes. TRAIL results in less communication overhead caused by sink mobility and is therefore very appealing for delay-tolerant networks. However for large scale networks, TRAIL would cause considerable data delivery latency for the farthest nodes as major part of the data delivery path adopted would be comprised of random walks.

To ensure uninterrupted data delivery to a mobile sink, a mobility prediction based scheme in proposed in [30]. The proposed scheme delivers sensory data to a mobile sink via relay nodes that are predicted based on the mobility graph of the mobile sink. The mobility graph is pre-computed based on a sequence of those nodes that receive relatively strong signals from the mobile sink. Each time, the mobile sink moves from one relay node to next, it floods the network with the information of the next predicted relay node and arrival time. This helps sensor nodes dynamically adjust their routes to the predicted next relay node in accordance with anticipated arrival time of the mobile sink. The reported experimental results reveal high packet delivery ratios at low speed of the mobile sink which gradually degrade as the speed increases. Furthermore, the proposed scheme considers sink mobility in an indoor office environment where the mobility is relatively constrained and thus easier to predict compared to an outdoor scenario.

To improve network lifetime and data delivery performance, a solution is proposed in [31] that is based on a three-tier network architecture. Tier-1 is formed by all the sensor nodes, while mobile sinks being working as data collectors form tier-2 and a static base-station forms tier-3. For data collection, predictable sink mobility is adopted and multiple mobile sinks approach the sensor nodes in different network segments thereby forming clusters around themselves. Consequently, mobile sinks query single-hop sensor nodes, collect data from them and report to the base-station. However, to avoid very long packet delivery latency and long waiting times, nodes forward sensory data towards an anticipated sink position. In case of large packet sizes, the proposed scheme splits packets into several smaller packets and delivers them to various mobile sinks simultaneously, assuming it will be reassembled at the base-station. Moreover, for guaranteed message delivery, the proposed scheme reduces sinks' velocity, thereby ensuring long contact time between sensor nodes and mobile sinks. The proposed scheme achieves improved network lifetime and high packet delivery ratios by employing multiple mobile sinks together with their slow and fixed speed. However, the reduced sink velocity results in increased data delivery latency.

The scheme proposed by Akkaya and Younis in [32], aims at balancing energy consumption and data delivery latency to a mobile sink. The proposed scheme minimizes the topological overhead caused by sink mobility and makes dynamic route adjustments. It adapts the network topology by exploiting the full/partial knowledge of the navigational map and the traveling schedule of the mobile sink. Accordingly, it assumes that the mobile sink moves in steps along a straight line to reach intermediate positions, where the size of the step is taken in accordance with the sink's speed. Initially, according to the primary position of the mobile sink, routes are set in an energy-efficient way to facilitate continuous data delivery till the sink moves to the next intermediate position. When it moves from one point to another, the network topology is reassessed. In the reassessment process, one of the following three choices is made: (1) the mobile sink is still within reach of the old neighbor node; (2) forwarder nodes are discovered; (3) rerouting is required. This process continues until the mobile sink arrives at a terminal position. Using the first choice, the sink instructs the last hop node to adjust its radio to cover the sink's next move if its residual energy is sufficient. When the residual energy is not enough, the sink exercises the second choice of appointing a forwarder. Finally, if no reasonable forwarder is available, then the sink exercises the last option to set up new routes. Rerouting is also triggered if the current paths to sink do not satisfy the end-to-end delay bound. A good feature of this scheme is that when the sink moves closer to its next stop, it can still overhear the communication between its new and old neighbor nodes and thus receives it directly. This results in reduced overall delay of that packet, thereby reducing the number of multi-hops. The main limitation of this scheme is the assumption that sink's navigational map and schedule are known, which may not be realized in many application environments.

The aforementioned path constrained-based data collection schemes are summarized in Table 1. It illustrates each scheme in terms of any constraints it imposes on sink mobility, the number of sinks involved for data collection, data reporting modes (proactive, reactive, query-driven, periodic, or wait-and-upload), network architecture, network overhead control mechanism, an estimate of network control overhead, together with the main goal(s).

### 7.2. Path Unconstrained Sink Mobility-Based Schemes

In this section, we discuss several data collection schemes where the sink moves autonomously and does not have any constraints on its trajectory. The sink can visit nodes anywhere in the sensor field for data collection. In the rest of this section, an overview of the state-of-the-art schemes is given, including their aims, methodologies, strengths and weaknesses.

Shi *et al.* proposed in [33] an efficient Data-Driven Routing Protocol (DDRP) for mWSNs which aims to reduce network control overhead in route discovery/maintenance and improve data delivery performance. In DDRP, the mobile sink exploits the broadcast feature of the wireless medium and periodically broadcasts beacon messages (containing sink ID, timestamp, and an optional variable beacon-interval field) to its one-hop neighbors as it moves around. In this way, the neighbor nodes come to know about the existence of the sink in their vicinity. These one-hop neighbors of the sink do not further propagate this beacon message, but rather each data packet carries an additional field, called *Dist2Sink* (Distance to Sink), which stores the shortest known distance (number of hops) to the sink using that node. For neighbor nodes of the sink, *Dist2Sink = 1* and for others, it might be *{2, 3, …., K}*, depending on their depth inside the sensing field. *K* corresponds to some maximum value to restrict the maximum hop-counts, beyond which *Dist2Sink = Infinity*, which means nodes with no routes to the sink. Nodes whose *Dist2Sink* is set to *Infinity*, wait for a certain amount of time to overhear about a valid route to the sink. However, if no overhearing is received within a time bound, nodes adopt a random-walk strategy till the packet finds a valid route to the sink or it is timed out and dropped. In addition to *Dist2Sink*, each data packet also carries *Time-Stamp* information (the time-instance when a route was created to a mobile sink) which is used to avoid routing loops. The main drawback of DDRP is that with each movement of the mobile sink, subsequent topological changes will occur that are propagated in the entire network in the form of the overhearing mechanism. The overhearing mechanism greatly compromises nodes' energy reserves [34] as nodes need to be in idle listening mode in order to overhear the sink's latest location.

Safdar *et al.* proposed a hybrid (proactive and reactive) routing protocol in [35] that basically enhances the IPV6 Routing Protocol for Low power and lossy networks (RPL) to efficiently support sink mobility. In the basic RPL, the mobile sink frequently advertises its presence through a broadcast mechanism propagated in the entire sensor field. In this way, the nodes, upon receiving these messages form a Directed Acyclic Graph (DAG) to the sink. Thus, a single move of the sink results in extensive network traffic and high energy consumption. In the hybrid approach [35], the sink's topological update is not propagated in the entire network, but rather it targets only a confined zone (consisting of a few hops) around the sink. Thus nodes within the confined zone can immediately send data to the sink using the known DAGs (proactive routing). For low sink mobility, zones with bigger sizes are created to facilitate prompt data delivery to the sink and for high sink mobility, zone sizes are kept smaller. Nodes that do not belong to any of the confined zones around any mobile sink exercise on-demand sink discovery (reactive mechanism). For that purpose, the nodes only broadcast route requests bounded by a few hops, thereby avoiding broadcasts to the entire network. Consequently, if this route request is received by any sink within that zone or any of the nodes that has an active DAG to the sink, it responds to the source node. The source node thus records that path for subsequent communications. However, using the first broadcast if the source node fails to receive a response, it waits for a while in the hope to find any nearest sink. At the expiry of that waiting time, it increases the zone's size for broadcasting the route discovery request. This procedure is repeated for some maximum number of retries, failure to which results into a network wide broadcast for route discovery. This scheme restricts the propagation of sink presence to a confined zone for achieving energy conservation, thereby avoiding network wide propagation. However nodes outside the confined zone have to find a route to the sink via the broadcast mechanism. Thus, during the sink discovery process, several broadcasts might need to be required depending on the position of the requesting node within the network. This will result into high energy consumption and collisions. Furthermore; using this approach, data delivery latency would also be more as a major part of the network has to establish routea to the sink first via the broadcast mechanism.

**Figure 8.**
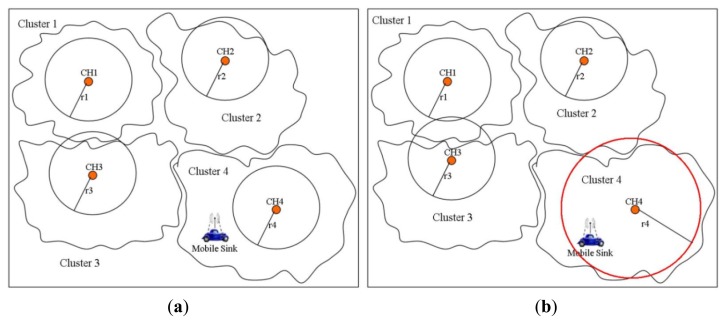
(**a**) Network Topology with default radio settings; (**b**) Network Topology after radio adjustment.

Tashtarian *et al.* proposed an energy efficient data gathering algorithm for cluster-based mWSNs [36]. Initially, it partitions the whole network into partially overlapped clusters and classifies the nodes as Cluster-Heads (CHs) and Cluster-Members (CMs). CHs collect data from their CMs according to some MAC schedule. For uploading the collected data to the mobile sink, first the CH checks whether the mobile sink is accessible within its default radio coverage range. However, if the sink is not reachable with the default radio configuration, the CH increases its transmission power in the hope to get to the sink as shown in Figure 8. This process continues until the CH finds the mobile sink in its new coverage area. At that point, the mobile sink comes to a stationary state until it receives all the transmitted data from that CH. This scheme simply replaces the multi-hop communication with a single long range communication irrespective of the constrained resources (limited energy reserve, short range radio) of the sensor nodes. The long range radio communication may also disrupt communication in other clusters thereby causing collisions followed by retransmissions which gives rise to more energy consumption and delays for the disrupted nodes. Moreover, it also does not incorporate sink mobility patterns and leaves this to the application's designer.

Integrated Location Service and Routing (ILSR) scheme is proposed in [37] that is based on geographic routing protocol and aims to ensure guaranteed packet delivery to a mobile sink. It assumes that all the nodes, including the sink, are aware of their location information. Furthermore, neighbor nodes also exchange their location information via *hello* messages. The mobile sink initially floods the network (once) with its location information and throughout the process remains connected via at least one node from that network. The sink moves slowly such that neighborhood change is detectable. To incorporate sink mobility, ILSR enhances the popular Geographical Forwarding Graph (GFG) routing protocol proposed in [38] which provides guaranteed delivery in static networks. ILSR enhances GFG with two types of location updates, namely *flooding-type* and *routing-type. Flooding-type* location updates target only the area near the sink in which the nodes detect some change in the next-hop node route towards the sink. Similarly, the *routing-type* location update targets the lost neighbors of the mobile sink to prevent routing failures in the network. Both these location update messages are time-stamped via a monotonically increasing sequence number for maintaining freshness. Furthermore, two versions of ILSR are proposed to deal with both unpredictable and predictable sink mobility. ILSR achieves guaranteed data delivery to the mobile sink via the two types of location update messages. However, this scheme is only applicable for delay tolerant applications because of the long convergence time for route adjustment in the case of sink mobility.

Inspired by the foraging behavior of termites, the Termite-hill routing scheme for WSN [39] aims to achieve better performance in terms of reducing control traffic overhead, fast route discovery and overall energy consumption. It assumes no prior knowledge about network configuration and adjacencies. The routes are selected based on energy utilization. Routes are discovered on demand, *i.e.*, when the nodes have to report some event. For route discovery to the sink, the node generates a *forward-soldier* packet which is broadcast to all neighbors. Upon receiving this packet, the intermediate node looks for a valid route to the sink in its routing table and if finds one, it generates and sends back a *backward-soldier* packet to the source node. However, if it does not have a valid route to the sink, it saves a reverse path to the source node (for future use) and continues broadcasting the *forward-soldier* packet. This process goes on until the *forward-soldier* packet gets to its destination (mobile sink). Accordingly, the sink, upon receiving this packet, sets up a *backward-soldier* packet and unicasts it to the source node. All the intermediate nodes upon receiving the *backward-soldier* packet modify their routing tables thereby setting up a forward pointer to the sink and forward the *backward-soldier* pack*et* along the reverse path hop-by-hop. This process goes on until the original source node receives the *backward-soldier* packet. Although this scheme is simple enough, it does not take into account the sink mobility pattern and the associated costs in terms of route discovery, latency and packet losses. It only investigates the potential benefits that are obtained by adopting a mobile sink for the sake of improving network lifetime.

**Figure 9.**
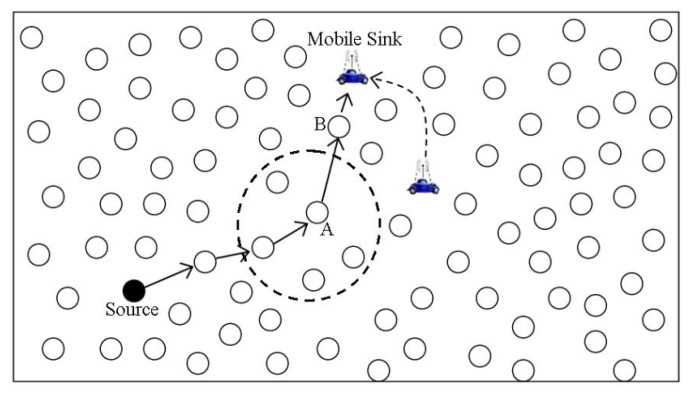
Mobile sink tracing.

To minimize the network control traffic due to sink mobility, Elastic routing was proposed by Yu *et al.* [7] that exploits the broadcast transmission nature of sensor nodes. To deal with sink mobility, the new sink's location is propagated along the reverse geographic routing path to the source node via the overhearing mechanism. It is claimed that the proposed solution results in reduced network control overhead, data delivery latency and energy consumption compared to other existing solutions dealing with sink mobility. It assumes that all nodes are location aware and hence share location information with neighbors via beacon messages. Furthermore, it assumes bidirectional channels between two neighbor nodes. In the initial phase, the source determines the sink location by some sink location service. For data forwarding, a source node first looks for a valid path to the sink in its neighborhood list, otherwise greedy forwarding approach introduced in [40] is adopted for route determination to the sink. However, before the forwarded data reaches the sink, the sink might have changed its position. To cope with this situation, Elastic routing proposes that along with periodic beacon messages to inform about new neighbor nodes, those nodes are also updated with the sink's latest location from which the sink has received the last packet. This phenomenon is illustrated in Figure 9, where the sink is unreachable by last hop *A*, so it informs its latest location to node *A* by greedy forwarding. Accordingly, node *A* updates the sink location information in the data packet and sets *B* as the next hop towards the sink. Elastic routing traces the sink mobility in this way, and thus ensures uninterrupted data delivery to mobile sinks. For further propagation of the latest sink location information in the network, elastic routing employs the overhearing feature of wireless sensor nodes. Since it assumes a bidirectional channel between two neighbor nodes, therefore whenever any neighbor node of the sink sends some data to the sink, it is overheard by other neighbor nodes in the vicinity. This overhearing process goes on step-by-step in the subsequent data forwarding till the source nodes comes to know the latest location of the mobile sink. Elastic routing proposes that upon overhearing, each node should compare its distance to the new sink location with the distance from the originator of transmission. Thus, if the originator distance to the sink is shorter than its own distance to sink, it caches the sink latest location information and otherwise it discards it. The main limitation of this scheme is the long convergence time which consequently results into long message delivery latency. Furthermore, it embeds the latest location information of sink in each data packet and exploits the overhearing mechanism for propagating the sink latest location information, which does not yield significant energy savings due to the idle listening.

In the case of uncontrolled sink mobility, Vecchio *et al.* proposed in [41] a Density-based proactivE data dissEmination Protocol (DEEP) that aims to obtain a representative view of the network sensed data by visiting only a subset of nodes. First, it addresses how to obtain an optimal number of nodes to be visited by the mobile sink while maintaining a good trade-off between the storage requirements on such nodes and the ratio between the visited nodes and the representativeness of the gathered data. The proposed scheme is based on proactive data dissemination, however, unlike other virtual structure-based data dissemination schemes such as those described in [12,23,27,42], the sensed data is not deposited in a subset of nodes to be retrieved later by a mobile sink. It is argued that depositing sensed data at nodes that form the virtual structure results into an imbalanced use of energy consumption and storage space as nodes forming the virtual structure and its one hop neighbors would quickly deplete their energy. Hence, to make a balanced use of energy consumption and storage space on nodes, it assumes that every sensor node is equipped with a buffer space that stores a partial view of the sensed data. The partial view is composed of not only the data sensed by the sensor node itself, but also a compressed version of its neighbors' observed data as well to be uploaded to a mobile sink. The proposed scheme avoids the network overhead associated with keeping track of sink mobility and keeps a partial local view of sensor nodes observed data to be retrieved by a mobile sink upon its visit to that segment of the network. However, it is only suitable for applications which are delay tolerant and where the rate of event generation is low.

**Figure 10.**
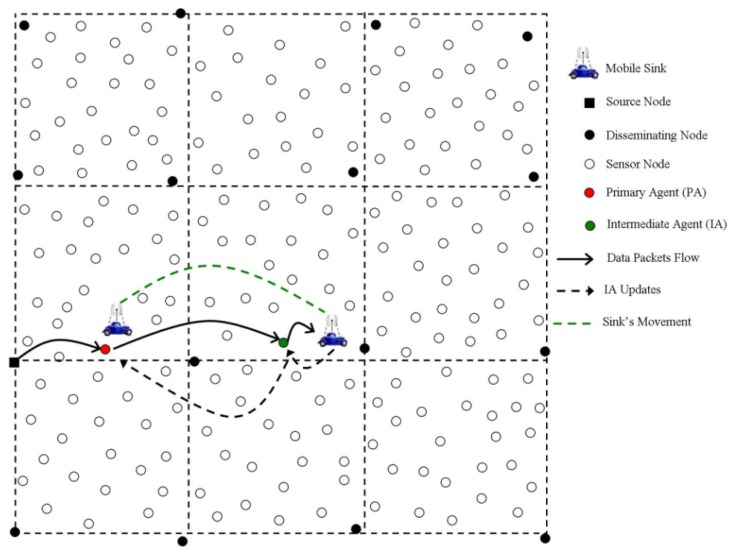
Handling sink mobility in TTDD.

The virtual grid-based Two-Tier Data Dissemination (TTDD) protocol for large-scale WSNs in [42] aims to minimize the flooding of sink topological updates. This protocol periodically builds source/event-based virtual global grids (spanning the entire network area) where a data source node proactively constructs a virtual grid structure spanning the entire sensing field and arranges the forwarding information at all disseminating nodes (nearest nodes of grid points). A sink upon arrival at any cell of the grid structure, injects a query destined to the data source. The query utilizes the grid structure and traverses two tiers to reach to the data source: a lower-tier which is within the local cell of the sink's current location, and a higher-tier composed of all the disseminating points on the grid structure. Furthermore, each disseminating node is aware of its upstream and downstream team members. In traversal, the query first determines a nearest disseminating node by the flooding mechanism which is bounded by the size of the grid cell. From that point onwards, the query is relayed towards the source node along the upstream disseminating points until it arrives at the source node. Accordingly, the source sends the response message along the reverse path of the query message. To cope with mobility, the sink appoints a Primary Agent (PA) node and an Intermediate Agent (IA) node. PA is the nearest node to the sink's current position and collects data from intermediate disseminating nodes to deliver it to the sink. Each query message contains location information about the PA. Initially both the PA and IA are the same. Upon changing its location, when the sink is about to lose the IA (also the PA initially) due to coverage issues, the sink appoints another IA and updates the PA about the new IA's location for directing the future data towards the new IA. This phenomenon has been illustrated in Figure 10. Similarly, if the sink moves over a considerable distance (such as a cell size) from its PA, it repeats the aforementioned process by flooding the query bounded by cell size to find new disseminating node and accordingly chooses a PA for that new intermediate disseminating node. The proposed scheme achieves considerable energy savings thereby avoiding the flooding of the whole sensing field. However, the cell size needs to be set carefully as it has a direct impact on the size of the flooding area and grid construction/maintenance, *i.e.*, a larger cell size means a wider flooding area, whereas a smaller cell size gives rise to more overhead involved in the grid construction/maintenance. Furthermore, constructing a grid for every single source/event on a periodic basis results in more energy consumption [43]. In addition, in case of sink mobility, this scheme just ensures the uninterrupted data delivery, but the route adopted might not be the optimal route (number of hops involved), thereby potentially compromising the data delivery latency.

**Figure 11.**
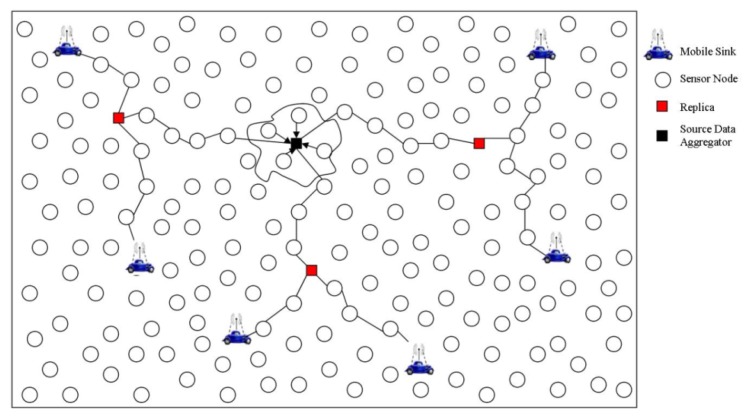
Data dissemination tree model in SEAD.

A Scalable Energy-efficient Asynchronous Dissemination (SEAD) protocol has been proposed in [44] that aims to prolong the network lifetime by employing multiple sinks. SEAD constructs a dissemination tree (called *d-tree*) both for routing and caching data. Any mobile sink interested in communicating with the source of the tree joins the *d-tree* by sending a *join-query* to one of its neighbor sensor nodes. This neighbor node becomes the sink's access-node and is responsible for data delivery to the sink. The access-node upon receiving the *join-query*, recursively constructs a *d-tree* by exploiting the geographic location information. The access-node also keeps track of sink mobility. The *d-tree* is updated whenever the sink changes its access-node. However, to avoid frequent reconstruction of the tree caused by sink mobility, the sink does not choose another access-node until the hop-counts between sink and access-node exceed a certain threshold. When a new access-node is selected, the old access-node is informed accordingly. This strategy is adopted to maintain the trade-off between energy-expenditure in the tree-reconstruction and path-delay. Furthermore, to disseminate data to multiple sinks, a set of nodes between the source and sinks are chosen as replica that keep the source data temporarily. An example SEAD tree model is shown in Figure 11.

SEAD achieves improved energy consumption, thereby avoiding frequent re-construction of the *d-tre*e. However, in pursuit of energy conservation, it incurs additional delays (in the form of a few extra multi-hops towards the sink's latest position) and is therefore not applicable for delay-sensitive applications. Furthermore, to maintain the *d-tree*, if a source has no more sensory data to report, it still sends idle messages to sinks, which results in unnecessary energy consumption.

The aforementioned data collection schemes which do not impose any constraints on the path of the mobile sink(s) are summarized in Table 2. It illustrates each scheme in terms of any constraints it imposes on sink mobility, the number of sinks involved for data collection, data reporting modes (proactive, reactive, query-driven, periodic, or wait-and-upload), network architecture, network overhead control mechanism, an estimate of network control overhead, together with the main goal(s).

### 7.3. Controlled Sink Mobility-Based Schemes

In this category, those data collection schemes where the sink mobility is under the control of the network and/or observer of the network to achieve some specific goal are discussed. The goal highly depends on applications where in most of the cases it is improving the network lifetime, while some focus on improving packet delivery ratio or reducing data delivery latency to meet real-time communication requirements. In the rest of this section, an overview of the various proposed schemes based on controlled sink mobility is given, including their aims, methodologies, strengths and weaknesses.

Aioffi *et al.* proposed a virtual structure-based data dissemination scheme for efficient data collection [45] using a mobile sink. The proposed scheme aims to reduce message delivery latency thereby optimizing trade-off between network lifetime and delivery latency. To do so, it adopts optimization algorithms (ICRP [46] and ILS [47]) to define optimal density control policies, sensor clustering and sink routes. It imposes topologic constraints (sensor clustering) to reduce energy consumption and to avoid collisions, thereby allowing only CH nodes to communicate with mobile sinks when the sinks arrive at the CH positions.

It integrates the clustering and routing problems together, thereby providing a virtual infrastructure (all CH positions) for the subsequent visits of the mobile sinks. The idea behind clusters formation is to minimize the set of nodes that need to be visited by the mobile sinks, thus resulting in reduced message delivery latency along with less energy consumption due to the shorter paths to the sinks. After clustering, it next determines a set of routes for mobile sinks such that every single sensor node is either being covered by that route, or lies within the communication range of another CH in another route. During sink movement, each sink broadcasts a message every second to alert the nearby nodes to be prepared for communication. Sensor nodes upon receiving the alert and coming in contact with mobile sinks forward their stored data (if any).

To avoid collisions if there are too many transmissions, the process is interrupted and a schedule is adopted when the mobile sinks arrive at the CHs. The proposed scheme reduces message delivery latency by employing several mobile sinks operating simultaneously in various segments of the sensor field thereby reducing the waiting time of nodes to deliver their sensory data to the sinks. However, the use of multiple mobile sinks limits its applicability to certain applications only. Furthermore, direct communication of the mobile sinks with the sensor nodes or only via the single-hop CHs may not be feasible in certain situations such as in battlefields or forest environments.

**Figure 12.**
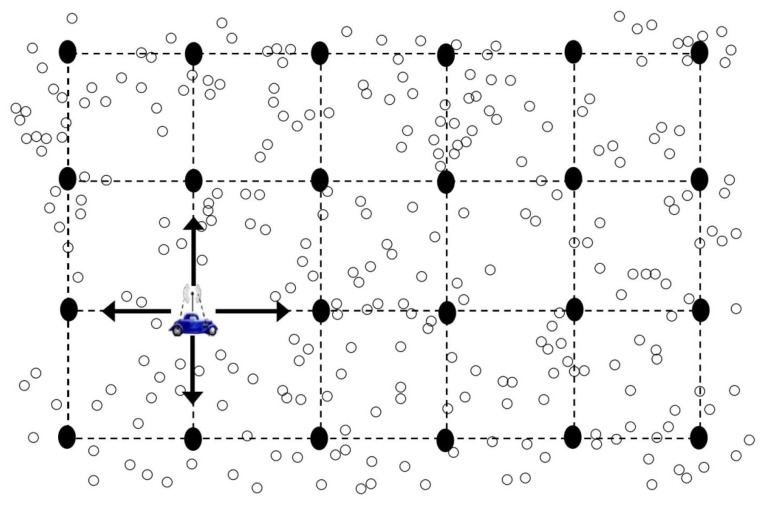
Example of overlay graph G_0_ for network traversal [12].

Kinalis *et al.* proposed a method for energy-efficient and latency-aware data collection in mWSNs that exploits biased sink mobility with adaptive stop times [12]. It is based on the idea that due to sink mobility, certain nodes may not complete their data reporting to the sink and thus have to wait for the sink's next trip. This results in high data delivery latency and potential message losses. This problem becomes severe if the nodes density in an area is high or if the nodes have a significant amount of recorded data [12]. To alleviate the short stay problem of the mobile sink in a densely deployed area, this scheme introduces an adaptive pause time proportional to local data traffic. To do so, it requires knowledge about global network resources such as the initial energy reserves of the network and thus information about the expected network lifetime. Moreover, it assumes that in certain areas more sensors are deployed compared to other areas for fine grain monitoring purposes. Thus, it assumes that few pockets (areas with high node density) exist in the network. Accordingly, during sink movement, it makes relatively large pauses at regions with high node density. For network traversal, it assumes that a graph is formed during the network initialization phase which results in a lattice graph G_0_ = G(V,E) overlayed in the network area as shown in Figure 12.

To support different application scenarios, it employs both deterministic-walk and biased-random-walk mobility patterns for network traversal. In the deterministic-walk, the sink traverses every single cell thus covering the whole network area. In certain scenarios, where network topology is not known to the sink, a random walk mobility pattern is adopted. Furthermore, to provide fair coverage to different areas of the network, biased-random-walk mobility is thereby adapted at the end of every stop period, less frequently visited regions are favored for the next move of the sink by keeping a record of visits of every vertex.

The scheme proposed in [12] achieves relatively better performance in terms of reducing latency by introducing the adaptive pause time mechanism. Although the adaptive pause time mechanism is beneficial for the current network segment, it may incur large latencies in data delivery from other segments of network due to the increased pause time of the sink.

To address the hot-spot or energy-hole problem, Nazir and Hasbullah proposed a Mobile Sink based Routing Protocol (MSRP) in clustered WSNs [48] aiming at prolonging the network lifetime. Within a cluster, a CH performs data collection from its member nodes and waits for the arrival of the mobile sink. Whenever a mobile sink moves, it broadcasts a TDMA schedule to all the nearby CHs in its coverage area. Each CH will follow the schedule to forward its deposited data to the mobile sink. The decision about the next CH to be visited is taken based on the residual energy of the CH and for that purpose, the mobile sink keeps a record of the residual energy of all the CHs. Thus, it favors movement towards energy rich zones and consequently, moves towards the CH having more residual energy. A mobile sink collects data not only from CHs, but also from other neighbor nodes while moving. In this way, a balanced use of the node's energy results and this avoids the hot-spot problem thereby prolonging the overall network lifetime. This approach manipulates the next move of the sink towards energy rich zones in the network for improving network lifetime. However, it does not take into account the data delivery latency and the associated packet losses due to sink mobility. Using this approach, the sink will always move towards energy rich zones, thereby causing very huge data delivery latency from those parts of the network where some events are occurring most frequently and thus their residual energy will be relatively less.

**Figure 13.**
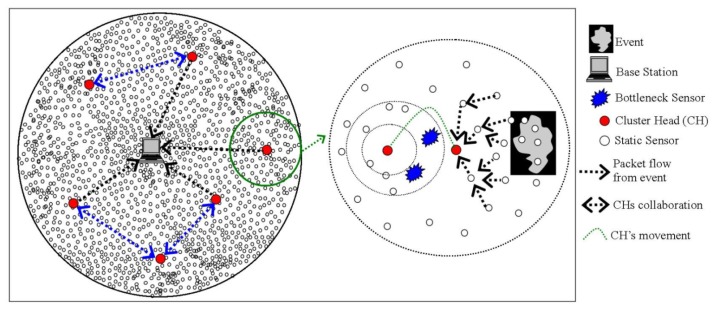
Multiple cluster-heads based mobility model [49].

To prolong network lifetime while ensuring the delay requirements of real-time applications, Banerjee *et al.* employ multiple energy-rich mobile CHs in [49], where the mobile CHs work in a collaborative manner to collect data from different segments of a network and deliver it to a base-station. The base-station, being static, is situated at the centre of the sensing field and receives data from the CHs within its radio range. This configuration is shown in Figure 13. To incorporate real-time application requirements, all the CHs are moved in a collaborative manner ensuring their connectivity with the base-station while covering most likely event reporting nodes at the same time. Furthermore, CHs are moved using three different strategies to reduce multi-hop communication and improve nodes' and network lifetime. In the first strategy, CHs are moved towards those sensor nodes where the sensor nodes have relatively more residual energy. This approach is inspired by the fact that an event that occurs is likely be sensed by a group of nearby sensor nodes, thus leading to a spatially distributed energy dissipation. In the second mobility strategy, CHs move towards the event region indicated by more traffic flow. This strategy is adopted to reduce the expected transmission and reception time, thereby reducing the distance between the CHs and the event source. Finally, in the third strategy, a hybrid approach is adopted, thereby first selecting to move the CHs towards the event source and while making the next move towards the source of event, the residual energy-based mobility strategy is being adopted. The experimental results produced in [49] show an increase of up to 75% in the residual energy by employing multiple mobile CHs and controlling their movements using the aforementioned strategies. However, the use of multiple energy-rich mobile CHs and the associated cost limits the widespread applicability of the proposed strategies.

Another contribution towards prolonging network lifetime exploiting controlled sink mobility has been made by Basagni *et al.* in [50]. In the proposed approach, few considerations are made such as how the next move of the sink will affect the latency of data delivery to the sink and the energy consumption of the nodes in adjusting the routes towards the mobile sink. It is argued that usually during sink movement, not only the data delivery latency is increased as packets need to be buffered at that time, but also the energy consumption in readjusting the routes due to sink mobility. Thus in order to control the rate of sink mobility, a minimum pause interval is imposed at various sites. For sink mobility, it defines a heuristic called Greedy Maximum Residual Energy (GMRE) which dictates sink mobility towards energy rich sites, but in accordance with the cost of data route release and establishment due to sink movement to that site. Consequently, if the site is an area that has relatively high energy than the current site and the route control overhead is not significant, the sink will greedily move to that new site, otherwise it will stay at its current site. To know about the residual energy of nodes in adjacent sites, the sink appoints a sentinel node responsible for collecting residual energy of nodes in its site. The sentinel node whenever asked by mobile sink responds with the collected information. The controlled sink mobility approach results in network-wide balanced energy consumption and hence improves the network lifetime. It is claimed that this controlled mobility results in 50% to 100% longer network lifetime compared to random uncontrolled mobility. The main limitation of this scheme is that the less energy areas would always be neglected and would cause huge latency for data delivery to the mobile sink. Furthermore; to maintain energy profiles of different areas of network, a considerable amount of node's energy reserve would be consumed as nodes have to report their residual energy level whenever requested.

The Anchor-based Voronoi-scoping Routing Protocol (AVRP) [29] proposed for moderate to high traffic load mWSNs aims to reduce communication overhead and improve data delivery performance. It employs multiple mobile sinks where each sink collects data via anchor nodes from a subset of sensor nodes located in its Voronoi cluster. The anchor nodes act as immediate sinks for nodes within Voronoi clusters and are dynamically selected based on signal strength measurements by the mobile sinks to cope with sink mobility. Once a new anchor node is selected, the mobile sink floods its interest to nodes located within its Voronoi scope via anchor node. Each interest packet contains the sink's ID, anchor's ID, and time-stamp information, together with hop-count distance to the anchor node. Nodes within the Voronoi scope upon receiving an interest packet compare these entries against their local route table and update their record if necessary, otherwise discard the interest packet. By employing multiple mobile sinks, AVRP localizes communication overhead caused by sink mobility within the Voronoi scope of the sink. However, each time the sink moves and selects a new anchor node, more interest packets need to be flooded within the Voronoi scope of mobile sinks. Depending on the sink's speed, frequent movements trigger more flooding of interest packets which potentially undermines network lifetime.

Sugihara and Gupta in [51] proposed a scheme to improve data delivery latency by using a controllable data mule (mobile element) so that data can be collected from sensor nodes in the shortest amount of time. The use of data mules is considered as a substitute for multi-hop forwarding and is being exploited in several schemes such as [5,17,52–55]. In the solution proposed by Sugihara and Gupta, it first tries to find such a path for scheduling the motion of the data mule so that data from all sensor nodes is harvested in less travel time. To do so, first, it finds the Travelling Salesman Problem (TSP) tour *T*. Then, by using an approximation algorithm, it applies shortcutting to *T* in order to obtain shortest label-covering tour. In a single trip, the data mule harvests data from all sensor nodes using single-hop communication. In the proposed scheme, the controlled motion of the sink reduces the overall data delivery latency by making use of direct single-hop communication; however, the single hop communication with the sink might not be realizable in many application environments such as forests, battlefields. *etc.* Furthermore, for large scale networks and dense random deployment, traversing all the sensor nodes in a single tour without redundantly visiting some nodes becomes infeasible.

In [56], Kotsilieris and Karetsos proposed a clustering mechanism that exploits multiple mobile relays to prolong the network lifetime. Basically, the proposed mechanism extends the clustering approach introduced in [57], to address the energy-hole problem in the vicinity of the fixed cluster-heads/relay nodes. The proposed scheme initially partitions the network into clusters where in each cluster the relay-nodes perform data collection and coordination. The relay nodes are considered rich in resources and perform direct communication with a static sink. Furthermore, the relay nodes move to new locations within the formed clusters such that the overall energy consumption is minimized. To relocate the relay node to a new position, the proposed scheme considers all those locations that are not farther than a maximum distance from all member nodes within that cluster. This constraint is imposed to preserve the already formed cluster topology. Once such location is found, the relay node is moved to that location for better energy savings. Although, the proposed scheme improves the network lifetime by virtue of relocating relay-nodes; however, the extra cost incurred due to the use of multiple mobile relay nodes limits its applicability.

Network-Assisted Data Collection (NADC) is another controlled sink mobility scheme [58] that aims to prolong network lifetime while minimizing data delivery latency by adopting a Mobile Data Harvester (MDH). During data collection, the MDH follows an overlay graph comprising of two kinds of nodes namely Navigation Agents (NA) and Intermediate Navigators (IN). Both the NAs and INs form the sink's trajectory and assist the MDH in navigation through the sensor field. In order to reduce the data collection time and thus the data delivery latency, initially the Traveling Salesman Problem (TSP) approach is adopted such that in a single tour, the MDP covers all the NAs with least cost in terms of hop counts. Accordingly, those nodes which provide the shortest path among adjacent NAs become INs. Furthermore, all the single-hop neighbors of NAs are considered as Designated Gateways (DGs). The DGs are responsible to collect and buffer sensory data of their k-hop neighbors. For data collection, MDH sends a query message to the nearby NA asking about DGs, next NA and IA along its trajectory. NA accordingly provides the requested information and MDH collects buffered data from DGs on the way. The proposed solution prolongs the network lifetime with reasonable data delivery latency, but it is only applicable to small scale networks.

The MobiRoute routing protocol proposed by Luo *et al.* in [13] focuses on proactive routing towards a mobile sink where sensor nodes send their data in a multi-hop fashion on a periodic basis. It assumes the sink mobility pattern as a discrete mobility pattern in which the sink moves along several anchor points and makes temporary pauses at those points. The sink's pause time interval is much greater than the movement time between consecutive anchor points. According to authors, movement along the anchor points and the relatively large pause time, significantly reduce the overhead on part of the routing protocol thereby avoiding the need for frequent routes adjustments triggered by sink mobility. The sink while in transit establishes the link with the nearby nodes via beacon-messages. To avoid propagation of sudden and frequent topological changes of the mobile sink, MobiRoute propagates this up-to-date sink position information further in the network only when the sink arrives at an anchor point. This is because while in transit, the sink may come across multiple nodes and the links with them may no longer be valid when the sink arrives at next anchor point. To achieve improved network lifetime, MobiRoute also employs an adaptive mobility control mechanism thereby making energy-profiles of various network's segments. Based on the energy-profile, the sink avoids stopping at an anchor point if its energy-profile is extremely low. However, network-wide propagation of sink's topological updates triggered by a sink's arrival at a new anchor point results in extensive energy consumption. Alongside, another limitation of this scheme is the strict implication that the MAC protocol is overhearing energy-free, which may not a realistic assumption as performance-wise better MAC schemes such as B-MAC suffer greatly from overhearing problems [59].

Wang *et al.* exploit sink mobility for prolonged network lifetime in [60] where the mobile sink moves around a sensor field and makes temporary pauses at nodes placed at grid points. In the proposed scheme, sensor nodes are assumed to be placed deterministically at equal distances along the grid points, where the grid is considered as bi-directional having same-sized square cells. Furthermore, it assumes nodes have unlimited buffer size and communicate periodically with the sink in a multi-hop manner where one hop is considered as the cell size. The sink moves along the grid points to prolong the network lifetime. To do so, the sink makes a temporary pause at a particular node in accordance with the residual energy of that node. In order to determine the candidate nodes to be visited and the pause-period at those nodes, a linear optimization model is adopted with the aim of maximizing the network lifetime till first node in the network dies due to energy depletion. Compared to the static sink scenario, the proposed scheme improves network lifetime thereby providing balanced energy consumption among sensor nodes. However, the deterministic nodes deployment at equal distances and unlimited buffer-size assumption do not hold true in most sensor network deployments.

The aforementioned controlled sink mobility-based data collection schemes are summarized in Table 3. It illustrates each scheme in terms of any constraints it imposes on sink mobility, the number of sinks involved for data collection, data reporting mode (proactive, reactive, query-driven, periodic, or wait-and-upload), network architecture, network overhead control mechanism, and an estimate of network control overhead, together with the main goal(s).

## 8. Comparative Study

It is worthwhile to organize and evaluate the aforementioned data collection/dissemination schemes based on their primary goals. The majority of the aforementioned data collection schemes aims to prolong network lifetime by employing single or multiple mobile sinks and are listed in Table 4 in ascending order with respect to constraints they impose on network in order to operate. Similarly, Table 5 and Table 6 provide a comparative study of those data collection/dissemination schemes that address data delivery latency and successful data/packet delivery performance respectively. The idea behind this organization and comparative study is to enable readers interested in a particular goal to evaluate and select appropriate scheme in accordance with their applications and network dynamics. We evaluate and compare each scheme in terms of the following set of features:

*No. of Sinks*—The performance of a data collection strategy is greatly affected by the number of sinks employed for data gathering. Some schemes employ multiple mobile sinks (or one static sink and multiple mobile sub-sinks) operating in different segments of network at the same time, thereby reducing multi-hop communication [45,49]. The use of multiple sinks thus results into more energy savings and decreased data delivery latency. It also improves data delivery ratio as packets would be less vulnerable to be dropped due to expiry of TTL. On the downside, the use of multiple sinks not only increases the hardware and operating cost, but also requires tight collaboration among them in order to avoid redundant coverage of overlapped network segments. Another artifact is that if multiple sinks operate independently in the sensor field, it will cause more traffic congestion thereby increasing packet loss ratio and data delivery latency [42,44].
*Sink Assistants*—Some of the existing schemes make use of several sink assistants. Sink assistants are also considered as less-constrained in terms of resources (energy, storage) and can be either static nodes or mobile entities. In static scenarios, these assistant nodes adopt the responsibility of local data collectors to be approached at a later stage by a mobile sink [23,27,28]. In mobile scenarios, they provide simultaneous coverage of various network segments to provide real-time communication services and reduce nodes energy consumption [49,56].
*Sink Mobility Pattern*—The mobility pattern of a sink greatly affects the data dissemination process. As mentioned before, sink mobility can be random [7,33,36,37,39,41,42,44], predictable/deterministic [23,25–28,32], and/or controlled [12,13,48–51,56]. Sink mobility pattern is dictated by application environment (hostility, terrain), nodes density, and species (animal, human, robot) to which the sink device is attached/mounted.
*Sink Movement Type*—A mobile sink either moves continuously or exhibits discrete mobility thereby making temporary pauses. Many existing data collection schemes impose discrete mobility [12,13,25,32,35,36,42,48–50,56] in order to reduce network control overhead due to sink mobility and improve data delivery performance.
*Sink Speed*—It highly depends on to what entity (animal, human, car, airplane) the sink device is being attached. A mobile sink can either move at fixed or variable speed and can be considered as slow (up to 1 m/s), moderate (1 to 20 m/s), or fast (greater than 20 m/s). Sink's speed also affects the performance of data dissemination mechanism; for instance, slow movement of the sink would cause huge delay in data delivery for the father reporting nodes. On the other hand, using a very fast sink would result in frequent link breakages and establishments.
*Network Size*—Network size is considered as the total number of nodes in the network. It is highly application dependent and has a direct impact on performance of data delivery to sink. For convenience, here we consider network size as small (up to 100 nodes), medium (100 to 400 nodes), or large (greater than 400 nodes). In the applications where network size is small, controlled sink mobility yields greater energy savings [51].
*Location Awareness*—It specifies whether in the considered scheme sensor nodes are aware of their geographical coordinates or not. In routing mechanisms, especially location-based routing, nodes' location information can be helpful in a number of ways: First, if destination's (sinks') position is known, significant energy savings can be achieved thereby avoiding energy expenditures in route discovery [61]. Secondly, nodes do not need to store routing tables, thereby saving considerable memory space [62]. Thirdly, nodes' location information can be quite helpful in preserving the size of clusters and topologies [63], cluster formation and in cluster-head selection [64]. Similarly, query and response packets can be quickly propagated to destinations based on appropriate next hop selection [65]. Finally, in case of mWSNs, nodes' location information can be used to restrict propagation of sinks' topological updates to a limited number of nodes, e.g., recently visited nodes, avoiding frequent topological updates if mobile sinks' location and arrival time can be predicted as in [66], and avoiding duplicate visits in a single tour of a mobile sink [45]. However, to determine location information, it either requires nodes to be equipped with specialized hardware (GPS) or needs to run dedicated localization algorithms. The use of GPS not only incurs additional hardware costs but it is also energy consuming, whereas the localization algorithms which make use of beacon nodes consume considerable amount of energy during the message exchange process.
*Data Reporting Mode*—Data can be disseminated to a mobile sink either using any one of the five modes (or in combination with others): wait-and-upload, proactive, reactive, periodic-basis (time-driven), and query-driven. In wait-and-upload approach, sensor nodes buffer sensory data till a mobile sink arrives in their vicinity for data collection [12,45,48,51]. A proactive approach is adopted when paths to destinations are already known to reporting nodes and thus the observed sensory data is proactively disseminated either to sinks [7,13,32,37,56] or stored temporarily at some specific nodes in the network to be retrieved at a later stage by a mobile sink [23,28,41]. In the reactive approach, paths to sinks are not known *a priori* and are determined and used on demand whenever an event of interest occurs [36,37,39]. In the periodic approach, sensor nodes perform the sensing and data dissemination tasks on a periodic basis [33,36]. Query-driven data dissemination is exhibited when sensor nodes receive a query from sink asking for required data [25,27,44].

## 9. Conclusions and Future Works

In this paper, we have provided an extensive literature review of various existing schemes that exploit sink mobility to alleviate the energy-hole problem and thus improve network lifetimes. Based on sink mobility patterns, we have classified the existing mobile sink-based data collection/dissemination schemes into path constrained, path unconstrained, and controlled sink mobility schemes. It is observed that most of the existing schemes exploit sink mobility to prolong the network lifetime while few focus on data delivery latency and reliability (successful packet delivery) issues which are caused due to the dynamic topology of sink mobility. Furthermore; it is observed that the existing schemes mitigate data delivery latency by either: (1) employing multiple mobile sinks covering various segments of sensor field simultaneously; or (2) explicitly controlling sink mobility (speed and direction) to reduce multi-hop communication in data delivery; or (3) deploying super-nodes which are rich in energy and/or storage-capacity to work as access-points to the mobile sinks. We argue that the use of multiple mobile sinks for reducing data delivery latency requires tight collaboration among the multiple mobile sinks and violates the intended low-cost nature of WSNs. Similarly, explicitly controlling the sink mobility so that the sink visits nearly every single sensor node and harvests data directly in less time may not be a viable option in many harsh environments such as battlefields or forests. Moreover, deterministic deployment of super-nodes to work as access-points for mobile sinks is against the basic theme of WSNs (low-cost and self-organized nature). In addition, neighbor nodes of these super-nodes may potentially suffer from more energy consumption compared to distant nodes thus resulting in energy-holes in the network. Some of the existing solutions are very promising for reducing data delivery latency, but are applicable only in query-driven applications. An ideal and generalized solution in case of mWSNs should cover data dissemination both in query-driven and event-driven (reactive) modes. As part of future work, we plan to incorporate cluster- based data dissemination together with mobility-aware duty-cycle mechanisms not only to further optimize the trade-off between energy consumption and data delivery latency, but to improve packet delivery ratios at the same time. We are interested in introducing sink mobility prediction in data routing and then integrating the routing and MAC mechanisms. Our approach will be based on sink mobility prediction and dynamic scheduling so the duty-cycles and time-slots of the nodes would be adjusted in accordance with the data delivery path learned from mobility prediction algorithms.

## Figures and Tables

**Table 1.** Summary of path constrained based data collection schemes.

**Data Collection Scheme**	**Sink Mobility Constraint**	**No. of Sinks**	**Data Reporting Mode**	**Network Architecture**	**Overhead Control Mechanism**	**Estimated Network Overhead**	**Goals**
**MESS [23]**	Slow speed of sink	Single	Proactive	Heterogeneous nodes deployment	using multiple static sub-sinks	Low	Balancing network lifetime and latency
**VCCSR [25]**	Motion along a circular path outside sensor field	Single	Query Driven	Homogeneous nodes deployment	All the cluster-heads constituting the virtual backbone are informed only	Medium	Prolonging network lifetime
**Whisper [26]**	Constant speed along a straight line	Single	Query Driven	Homogeneous nodes deployment	Assuming no change in speed and direction of sink	Low	Guaranteed delivery to high speed sink
**[27]**	Motion along periphery of sensor field	Single	Query Driven	Heterogeneous nodes deployment	Through targeted query messages	Low	Reducing data delivery latency
**[28]**	An offline set of possible trajectories are used	Multiple	Proactive	Heterogeneous nodes deployment	Informing nodes about a set of all possible trajectories (Once)	Medium	Guaranteed data delivery
**TRAIL [29]**	Constant speed	Multiple	Reactive	Heterogeneous nodes deployment	Informing only single-hop neighbors	Low	Improving network lifetime
**[30]**	Motion in indoor office environment	Single	Proactive	Homogeneous nodes deployment	Triggering updates only upon transition from relay nodes	High	Improving data delivery performance
**[31]**	Constant speed and direction in each epoch	Multiple	Query-Driven and Wait-and-Upload	Homogeneous nodes deployment	Not specified	Low	Improving data delivery and network lifetime
**[32]**	Slow motion along stride	Single	Proactive	Homogeneous nodes deployment	Assuming nodes have partial/full knowledge of sink's navigation map and schedule	Low	Balancing network lifetime and delivery latency

**Table 2.** Summary of path unconstrained based data collection schemes.

**Data Collection Scheme**	**Sink Mobility Constraint**	**No. of Sinks**	**Data Reporting Mode**	**Network Architecture**	**Overhead Control Mechanism**	**Estimated Network Overhead**	**Goals**
**DDRP [33]**	No constraints	Multiple	Periodic	Homogeneous nodes deployment	Overhearing mechanism	Low	Prolonging network lifetime
**[35]**	No constraints	Single	Proactive and Reactive	Homogeneous nodes deployment	Nodes within a confined zone around the sink are only informed	Medium	Prolonging network lifetime
**[36]**	No constraints	Single	Reactive	Homogeneous nodes deployment	Not Specified	Medium	Prolonging network lifetime
**ILSR [37]**	No constraints	Single	Proactive	Homogeneous nodes deployment	Nodes within a confined zone around the sink are only informed	Low	Guaranteed message delivery
**[39]**	No constraints	Single	Reactive	Homogeneous nodes deployment	Not Specified	Medium	Prolonging network lifetime
**[7]**	No constraints	Single	Proactive	Homogeneous nodes deployment	Overhearing mechanism	Low	Guaranteed data delivery with minimal network control overhead
**DEEP [41]**	No constraints	Single	Proactive	Homogeneous nodes deployment	Not Specified	Low	Improving network lifetime while minimizing the hardware cost
**TTDD [42]**	No constraints	Multiple	Query driven and Proactive	Homogeneous nodes deployment	Sinks update only old neighbors	Medium	Improving network lifetime
**SEAD [44]**	No constraints	Multiple	Query driven	Homogeneous nodes deployment	Sink updates only old neighbors	Medium	Improving network lifetime

**Table 3.** Summary of controlled sink mobility based data collection schemes.

**Data Collection Scheme**	**Sink Mobility Constraint**	**No. of Sinks**	**Data Reporting Mode**	**Network Architecture**	**Overhead Control Mechanism**	**Estimated Network Overhead**	**Goals**
**[45]**	Visiting only a set of cluster-heads	Multiple	Wait-and-Upload	Homogeneous nodes deployment	Triggering updates only when sink arrives at any cluster-heads	Medium	Balancing network lifetime and latency
**[12]**	Discrete mobility pattern	Single	Wait-and-Upload	Homogeneous nodes deployment	Not specified	Low	Balancing energy consumption and delivery latency
**MSRP [48]**	Movement towards energy rich areas	Single	Wait-and-Upload	Homogeneous nodes deployment	Not specified	Medium	Prolonging network lifetime
**[49]**	Movement towards energy rich areas or event source	One static sink and multiple mobile sub-sinks	Periodic	Heterogeneous nodes deployment	Bounded by area covered by each mobile sub-sink	Low	Improving network lifetime while delivering real-time data
**GMRE [50]**	Discrete mobility pattern	Single	Periodic & Proactive	Homogeneous nodes deployment	Imposing a minimum pause interval at each sojourn point	Low	Prolonging network lifetime and delay bound delivery
**AVRP [29]**	Constant speed	Multiple	Proactive	Homogeneous nodes deployment	Bounded by Voronoi scope of each mobile sink	Medium	Improving data delivery performance
**[51]**	Following a computed trajectory covering all senor nodes	Single	Wait-and-Upload	Homogeneous nodes deployment	Not specified	Medium	Reducing Data Delivery Latency
**[56]**	Relocating within cluster boundary	Multiple mobile relays	Proactive	Heterogeneous nodes deployment	Not specified	Low	Improving network lifetime
**NADC [58]**	Motion along a computed trajectory	Single	Query-Driven and Wait-and-Upload	Homogeneous nodes deployment	Not specified	Low	Improving network lifetime while minimizing delivery latency
**MobiRoute [13]**	Discrete mobility pattern	Single	Proactive	Homogeneous nodes deployment	Imposing large pause interval	Low	Improving network lifetime
**[60]**	Discrete mobility pattern	Single	Periodic and Proactive	Homogeneous nodes deployment	Imposing large pause interval	Low	Prolonging network lifetime

**Table 4.** Comparative study of energy efficiency based data collection schemes.

Scheme	Performance Metrics							
	No. of Sinks	Sink Assistants	Sink Mobility Pattern	Sink Movement Type	Sink Speed	Network Size	Location Awareness	Data Reporting Mode
DEEP [41]	Single	No	Random	Continuous	N/A	Medium	No	Proactive
TRAIL [29]	Multiple	No	Random	Continuous	Moderate & Fixed	Medium	No	Reactive
MSRP [48]	Single	No	Controlled & Predictable	Discrete	N/A	Medium	No	Wait-and- Upload
NADC [58]	Single	No	Controlled & Predictable	Continuous	Moderate and Fixed	Small	No	Query-Driven and Wait-and-Upload
MobiR-oute [13]	Single	No	Controlled & Predictable	Discrete	Slow & Fixed	Small	No	Proactive
VCCSR [25]	Single	No	Predictable	Continuous	Fixed	Medium	Yes	Query Driven
TTDD [42]	Multiple	No	Random	Discrete	Moderate & Variable	Large	Yes	Proactive and Query Driven
DDRP [33]	Multiple	No	Random	Continuous	Moderate & Variable	Medium	No	Periodic
SEAD [44]	Multiple	No	Random	Continuous	Moderate & Fixed	Medium	Yes	Query Driven
[56]	Single	Yes	Controlled & Predictable	Discrete	N/A	Large	No	Proactive
[36]	Single	No	Random	Discrete	N/A	Small	No	Reactive
[39]	Single	No	Random	Continuous	Slow & Variable	Very Small	No	Reactive
[35]	Multiple	No	Random	Discrete	N/A	N/A	N/A	Proactive & Reactive
[60]	Single	No	Controlled & Predictable	Discrete	N/A	Medium	No	Periodic and Proactive

**Table 5.** Comparative study of data collection/dissemination schemes aiming at data delivery latency.

**Scheme**	**Performance Metrics**							
	**No. of Sinks**	**Sink Assistants**	**Sink Mobility Pattern**	**Sink Movement Type**	**Sink Speed**	**Network Size**	**Location Awareness**	**Data Reporting Mode**
[27]	Single	Yes	Predictable	Continuous	Variable	Large	Yes	Query Driven
GMRE [50]	Single	No	Controlled & Predictable	Discrete	N/A	Medium	Yes	Periodic & Proactive
[12]	Single	No	Controlled & Deterministic	Discrete	Moderate & Fixed	Medium	No	Wait-and-Upload
[51]	Single	No	Controlled & Predictable	Continuous	Moderate & Variable	Small	No	Wait-and-Upload
MESS [23]	Single	Yes	Predictable	N/A	Slow & Fixed	Small	No	Proactive
[45]	Multiple	No	Predictable & Controlled	Discrete	Slow & Fixed	Large	Yes	Wait-and-Upload
[32]	Single	No	Predictable	Discrete	Moderate & Variable	Small	Yes	Proactive
[49]	Single	Yes	Controlled & Predictable	Discrete	Slow & Fixed	Large	Yes	Periodic

**Table 6.** Comparative study of data collection/dissemination schemes aiming at successful data delivery performance.

**Scheme**	**Performance Metrics**							
	**No. of Sinks**	**Sink Assistants**	**Sink Mobility Pattern**	**Sink Movement Type**	**Sink Speed**	**Network Size**	**Location Awareness**	**Data Reporting Mode**
Elastic [7]	Single	No	Random	Continuous	Moderate & Variable	Large	Yes	Proactive
Whisper [26]	Single	No	Predictable	Continuous	High & Fixed	Large	Yes	Query Driven
ILSR [37]	Single	No	Random	Continuous	Moderate & Variable	Medium	Yes	Proactive
[28]	Multiple	Yes	Predictable	Continuous	High & Variable	Large	Yes	Proactive
[30]	Single	No	Predictable	Continuous	Moderate and Variable	Small	No	Proactive
AVRP [29]	Multiple	No	Controlled	Continuous	Moderate & Fixed	Medium	No	Proactive
[31]	Single	Yes	Predictable	Continuous	Slow and Fixed	Large	No	Query-Driven and Wait-and-Upload
